# A pre-ribosomal RNA interaction network involving snoRNAs and the Rok1 helicase

**DOI:** 10.1261/rna.044669.114

**Published:** 2014-08

**Authors:** Roman Martin, Philipp Hackert, Maike Ruprecht, Stefan Simm, Lukas Brüning, Oliver Mirus, Katherine E. Sloan, Grzegorz Kudla, Enrico Schleiff, Markus T. Bohnsack

**Affiliations:** 1 Centre for Biochemistry and Molecular Cell Biology, Georg-August-University, 37073 Göttingen, Germany; 2 Institute for Molecular Biosciences, Goethe University, 60438 Frankfurt, Germany; 3 MRC Human Genetics Unit, University of Edinburgh, Edinburgh EH4 2XU, United Kingdom; 4 Cluster of Excellence Frankfurt, Goethe University, 60438 Frankfurt, Germany; 5 Göttingen Center for Molecular Biosciences, Georg-August-University, 37073 Göttingen, Germany

**Keywords:** RNA helicase, small nucleolar RNA, ribosome biogenesis, RNA–protein complex

## Abstract

Ribosome biogenesis in yeast requires 75 small nucleolar RNAs (snoRNAs) and a myriad of cofactors for processing, modification, and folding of the ribosomal RNAs (rRNAs). For the 19 RNA helicases implicated in ribosome synthesis, their sites of action and molecular functions have largely remained unknown. Here, we have used UV cross-linking and analysis of cDNA (CRAC) to reveal the pre-rRNA binding sites of the RNA helicase Rok1, which is involved in early small subunit biogenesis. Several contact sites were identified in the 18S rRNA sequence, which interestingly all cluster in the “foot” region of the small ribosomal subunit. These include a major binding site in the eukaryotic expansion segment ES6, where Rok1 is required for release of the snR30 snoRNA. Rok1 directly contacts snR30 and other snoRNAs required for pre-rRNA processing. Using cross-linking, ligation and sequencing of hybrids (CLASH) we identified several novel pre-rRNA base-pairing sites for the snoRNAs snR30, snR10, U3, and U14, which cluster in the expansion segments of the 18S rRNA. Our data suggest that these snoRNAs bridge interactions between the expansion segments, thereby forming an extensive interaction network that likely promotes pre-rRNA maturation and folding in early pre-ribosomal complexes and establishes long-range rRNA interactions during ribosome synthesis.

## Abstract (precis)

This report explores the roles of the RNA helicase Rok1 in ribosome biogenesis. Several contact sites with 18S rRNA were identified which clustered in the “foot” region of the small ribosomal subunit. Additional analyses identified several new snoRNA–pre-rRNA base-pairing interactions.

## INTRODUCTION

**FIGURE 1.**
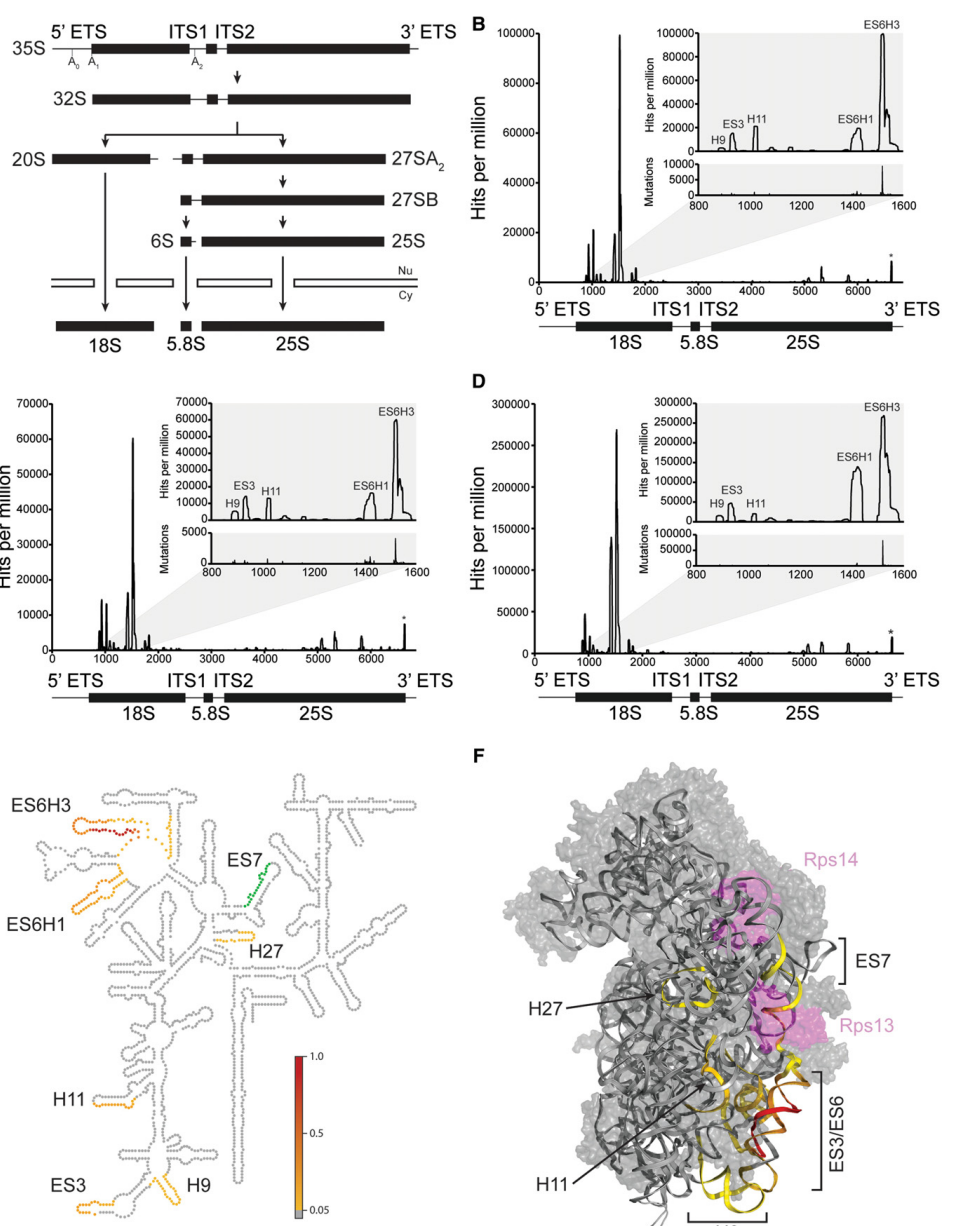
The Rok1 cross-linking sites on 18S rRNA cluster in the 3D structure of the small ribosomal subunit. (*A*) Schematic overview of the key steps in pre-rRNA processing pathway in *S. cerevisiae*. ETS, external transcribed spacer; ITS, internal transcribed spacer; Nu, nucleus; Cy, cytoplasm. (*B*–*D*) Cells expressing plasmid encoded N-terminally (*B*,*C*) or C-terminally (*D*) HTP-tagged Rok1 were depleted of endogenous Rok1 and UV cross-linked in vivo (*B*,*D*) or in culturo (*C*). Rok1-containing complexes were purified, cross-linked RNAs were trimmed, and linkers ligated, followed by RT-PCR and Illumina deep sequencing. Obtained sequence reads were mapped on the yeast genome and the results for RDN37 encoding the 35S pre-rRNA are plotted as number of hits per million reads for each nucleotide. Relative positions of the mature 18S, 5.8S, and 25S rRNA sequences are indicated *below*. A magnification of the major Rok1 cross-linking sites is given. Mutations arising during reverse transcription at cross-linking sites are shown in the *lower* panel of the zoom. The asterisk indicates a peak that is also present in untagged control samples. (*E*) Rok1 cross-linking sites (N-HTP in culturo) with hit values above a threshold of 5% of the highest peak on 35S rRNA are visualized as colored circles on the secondary structure of the mature 18S rRNA. Colors indicate peak height with the highest peak (100%) shown in red and lesser peaks shown in yellow. Nucleotides marked in green indicate a peak that was just above/below the 5% threshold in the different data sets. (*F*) Rok1 cross-linking sites (N-HTP in culturo) mapped on the 3D structure of the small ribosomal subunit. Peaks on rRNA are colored as in *E* and the location of cross-linking sites is indicated. H; helix; ES, expansion segment. Ribosomal proteins Rps13 and Rps14 are shown in purple.

The synthesis of cytoplasmic ribosomes in *Saccharomyces cerevisiae* is initiated by RNA polymerase I-mediated transcription of the 35S ribosomal RNA precursor (pre-rRNA), which contains the sequences of the mature 18S, 5.8S, and 25S rRNAs (Fig. 1A; Henras et al. 2008; Thomson et al. 2013; Woolford and Baserga 2013). This coincides with the recruitment of early ribosomal proteins to the nascent transcript and formation of the small subunit (SSU) processome from pre-assembled subcomplexes and individual biogenesis cofactors (for review, see Phipps et al. 2011). Initial cleavages (at the sites A_0_, A_1_, A_2_) (Fig. 1A) in a complex sequence of pre-rRNA processing and modification events lead to the separation of the biogenesis pathways of the large (LSU, 60S) and small (SSU, 40S) ribosomal subunits. In addition to 75 small nucleolar RNA–protein complexes (snoRNPs), which mediate both early pre-rRNA cleavages as well as most modification events, >200 protein cofactors are involved in ribosome production, among them 19 RNA helicases (Watkins and Bohnsack 2012; Martin et al. 2013; Rodriguez-Galan et al. 2013; Thomson et al. 2013). These RNA helicases have been proposed to act either in the structural remodeling of pre-ribosomal intermediates or in the unwinding of snoRNA–pre-rRNA base-pairing (Ripmaster et al. 1992; Venema and Tollervey 1999; Martin et al. 2013). Indeed, several RNA helicases are required for the release of individual snoRNAs from pre-ribosomes (Kos and Tollervey 2005; Liang and Fournier 2006; Bohnsack et al. 2008), and depletion of the RNA helicase Prp43 leads to the accumulation of several snoRNAs on pre-60S complexes (Bohnsack et al. 2009). However, the binding sites and molecular functions of most pre-ribosomal RNA helicases have remained elusive so far.

Based on conserved sequence elements (boxes), snoRNPs have been grouped into the box C/D snoRNPs that mostly catalyze 2′-*O*-methylation and the box H/ACA snoRNPs that can mediate pseudouridinylation of the rRNA (Watkins and Bohnsack 2012). Besides snoRNP functions in rRNA modification, several snoRNAs (U3/snR17, snR30, U14/snR128, and snR10) form base-pairing interactions with the pre-rRNA that are essential for the early events in pre-rRNA processing (see, for example, Morrissey and Tollervey 1993; Enright et al. 1996; Mishra and Eliceiri 1997). The snoRNA–pre-rRNA base-pairing directs the enzyme of the snoRNP complex to the specific target nucleotide and the interacting regions between the RNAs were recently found to be more extensive than previously thought (van Nues et al. 2011). Interestingly, modified residues cluster in the catalytic regions of the ribosome, such as the peptidyltransferase center, and neighboring modifications often require overlapping base-pairing sites of the snoRNAs that guide these modifications. This could imply that snoRNAs are recruited and function sequentially but it also raises the possibility that several snoRNAs may compete for base-pairing to common rRNA residues.

Here, we identify the binding sites of the DEAD-box RNA helicase Rok1 on pre-ribosomal RNA by UV cross-linking and analysis of cDNA (CRAC). Consistent with its role in small subunit biogenesis, we show that Rok1 interacts with several distinct sites in the 18S rRNA, which cluster at the “foot” of the small ribosomal subunit. The major Rok1 binding site is located in the eukaryotic expansion segment ES6, where Rok1 is required for the release of the essential snoRNA snR30. Rok1 also strongly associates with several snoRNAs, and by analysis of sequence hybrids we reveal a number of novel pre-rRNA base-pairing sites for the snoRNAs U3, U14, and snR10 on the 18S rRNA. Together, our data imply that, possibly along with various scaffold proteins, an extensive network of long-range snoRNA–pre-rRNA interactions might mediate the folding and structural remodeling of pre-ribosomal complexes.

## RESULTS AND DISCUSSION

### Rok1 cross-links to specific sites in 18S ribosomal RNA

To identify RNA interaction sites of the yeast RNA helicase Rok1 we made use of the CRAC method (Granneman et al. 2009; Bohnsack et al. 2012). Here, plasmid-encoded HTP-tagged Rok1 (N-HTP-Rok1 or Rok1-C-HTP) was expressed and confirmed to rescue the growth defects caused by depletion of endogenous Rok1 (Bohnsack et al. 2008), demonstrating that tagged Rok1 is able to functionally replace the genomically encoded Rok1. UV radiation was applied to cells pelleted from cultures (in vivo cross-linking; Bohnsack et al. 2009) or to actively growing cultures in medium (in culturo cross-linking; Bohnsack et al. 2012). The in culturo cross-linking method was established to reduce the manipulation of cells (e.g., by centrifugation, resuspension at high cell density) before cross-linking, in order to obtain cross-links under as near steady state conditions as possible. Rok1–RNA complexes were then isolated by tandem-affinity purification where the second step was performed under denaturing conditions to ensure that RNAs covalently cross-linked to, and therefore directly contacting, Rok1 would be isolated. Sequence libraries of bound RNA fragments were generated as previously described and analyzed by Illumina deep sequencing (Bohnsack et al. 2012). The sequence reads obtained were mapped on the yeast genome as previously reported (Bohnsack et al. 2009).

The distribution of Rok1 cross-linking sites on the primary 35S pre-rRNA transcript is shown in Figure 1B,D (in vivo cross-linking) and Figure 1C (in culturo cross-linking). The highest hit density (peaks) was found in the 18S rRNA sequence, consistent with the presence of Rok1 in early 90S and pre-40S pre-ribosomes and its role in the initial pre-rRNA processing events required for SSU formation (A_0_, A_1_, and A_2_) (Fig. 1A; Jakob et al. 2012; Lebaron et al. 2013; Segerstolpe et al. 2013). All three approaches showed a very similar distribution of hits across the pre-rRNA transcript, confirming that Rok1 cross-linking to these sites is unaffected by the position of the tag or the cross-linking method used. Within the 18S rRNA sequence, several prominent peaks between positions 800 and 1600 were readily detectable (zooms in Fig. 1B–D). Point mutations, introduced by the reverse transcriptase at nucleotides covalently linked to amino acid residues during UV cross-linking, were detected within all putative Rok1 binding sites in the 18S rRNA sequence (Fig. 1B–D zooms/lower graph), indicating direct RNA–protein contacts at these positions. Rok1 is the second RNA helicase, besides Prp43 (Bohnsack et al. 2009), for which binding sites were identified on yeast ribosomal RNA, and these two proteins show very different cross-linking patterns, indicating the specificity of the sites identified.

Mapping the Rok1 cross-linking sites onto the 2D structure of the mature 18S rRNA revealed that the main cross-linking sites of Rok1 are found in the eukaryotic expansion segment 6 (ES6) on helices (H) 1 and 3 (ES6H1 and ES6H3, respectively) (Fig. 1E). The other cross-linking sites of Rok1 are located in H11, eukaryotic expansion segment 3 (ES3), H9 that immediately precedes ES3, and H27 of 18S rRNA (Fig. 1E). In addition, cross-linking between Rok1 and sequences in eukaryotic expansion segment 7 (ES7) was observed in all experiments with peaks just above or just below the 5% threshold. Although the Rok1 cross-linking sites are distributed across the 5′ end of the 18S rRNA sequence, the formation of secondary and tertiary structures during ribosome synthesis can bring such distant regions of the pre-rRNA transcript together. We therefore mapped the cross-linking sites of Rok1 onto the 3D structure of the yeast 40S ribosomal subunit (Fig. 1F; Ben-Shem et al. 2011). Remarkably, all of the Rok1 cross-linking sites besides that in H27, which is only just above the threshold, clustered on one face of the “foot” region of the small ribosomal subunit (SSU). Furthermore, two ribosomal proteins, Rps14 and Rps13, have previously been shown to be required for Rok1 association with pre-ribosomes (Jakob et al. 2012). Interestingly, these proteins are localized adjacent to newly identified Rok1 cross-linking sites (Fig. 1F).

Taken together, the identification of Rok1–pre-rRNA cross-linking sites in the 18S rRNA sequence by our CRAC analyses is consistent with the role of this helicase in SSU maturation. Furthermore, the close proximity of the distinct Rok1 cross-linking sites in the 3D structure of the SSU might indicate that they are derived from a single Rok1 binding site on the pre-ribosome.

### Structure probing confirms Rok1 binding to ES6H3

**FIGURE 2.**
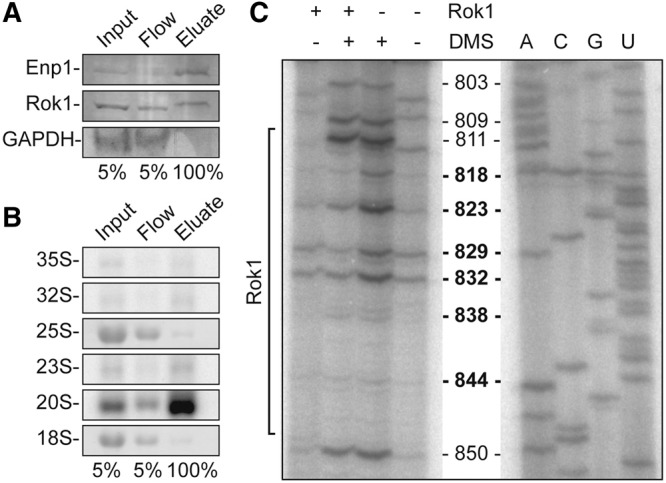
DMS structure probing confirms the interaction site of Rok1 in ES6H3 of the 18S rRNA sequence. (*A*) Extracts from cells expressing HA-tagged Rok1 under tetracycline control and Enp1-HTP were incubated with IgG sepharose and the eluate was analyzed by SDS-PAGE and Western blotting using antibodies against the HA-tag (Rok1), HTP-tag (Enp1), or endogenous GAPDH. (*B*) RNA from the Enp1-HTP pulldown experiments described in *A* was isolated and analyzed by Northern blotting using a probe hybridizing to the 5′ end of the internal transcribed spacer 1 (ITS1). Pre-rRNAs (indicated to the *left*) were visualized using a phosphorimager, and mature 18S and 25S rRNAs were detected by methylene blue staining. (*C*) Cells expressing Rok1 or depleted of Rok1 were treated with DMS in vivo and RNA that co-precipitated with Enp1-HTP (as in *A*) was isolated. RNA was analyzed by primer extension using a primer annealing downstream from ES6H3 to detect DMS-modified nucleotides (numbers indicate residues within the 18S rRNA sequence). Labelled RNA fragments were separated by denaturing polyacrylamide gel electrophoresis and visualized by phosphorimaging. The position of the Rok1 cross-linking site is indicated on the *left*.

To validate the pre-rRNA cross-linking sites of Rok1 identified here by CRAC we performed in vivo chemical probing (Wells et al. 2000), which allows us to detect structural changes within the predicted pre-rRNA interaction site upon Rok1 binding. Treatment of actively growing cells with DMS leads to methylation of accessible nucleotide residues (predominantly adenosines) and such modifications can be detected as primer extension stops. As the Rok1 cross-linking site is located within the mature 18S rRNA sequence, we isolated pre-rRNAs from Rok1-containing pre-ribosomal complexes to enable detection of changes in DMS-induced modification caused by Rok1 binding. Enp1 is an SSU biogenesis factor that is associated with both early and late pre-ribosomal complexes making it an ideal factor through which to isolate Rok1-containing particles. A yeast strain expressing genomically tagged Enp1-C-HTP was therefore used in pulldown experiments, and the presence of Rok1 as well as the enrichment of the 35S and 20S pre-rRNAs and lack of mature 18S rRNA were confirmed by Western and Northern blotting, respectively (Fig. 2A,B). RNA associated with Enp1 particles was isolated from DMS-treated cells either expressing, or depleted of, Rok1. Reverse transcription using a primer located downstream from the Rok1 cross-linking site in ES6H3 showed that the DMS modification of multiple nucleotides (such as A803, A809, A811, and A850) was unaffected by depletion of Rok1 (Fig. 2C). Excitingly, the accessibility of residues located within the Rok1 binding site (A817, G823, A829, and U832) was significantly increased (Fig. 2C). These data confirm that the Rok1 cross-linking site identified by CRAC represents bona fide interactions with the 18S rRNA sequence.

Interestingly, ES6H3 and sequences nearby were also recently identified as cross-linking sites of two other cofactors, Rrp5 and Rrp7, which are also involved in the early steps of ribosome biogenesis (Lebaron et al. 2013; Lin et al. 2013). While Rrp7 is recruited to the 90S complex as part of a pre-assembled module, the UTP-C complex, Rrp5 has further been proposed to function as a central scaffold, coordinating pre-ribosome assembly. Consistent with their adjacent pre-rRNA binding sites, Rrp5 has been shown to directly interact with Rok1. Furthermore, Rrp5 is suggested to modulate the activity of Rok1 and to confer target specificity on the RNA helicase (Garcia et al. 2012; Young et al. 2013). In the case of Rrp7, cross-linking sites in ES6H3 and ES7 that are overlapping with those suggested here by our Rok1 CRAC analysis were previously identified (Lin et al. 2013). Taken together, these data could suggest that Rok1 and Rrp7 may associate sequentially with pre-ribosomal complexes.

### Rok1 directly interacts with snR30

**FIGURE 3.**
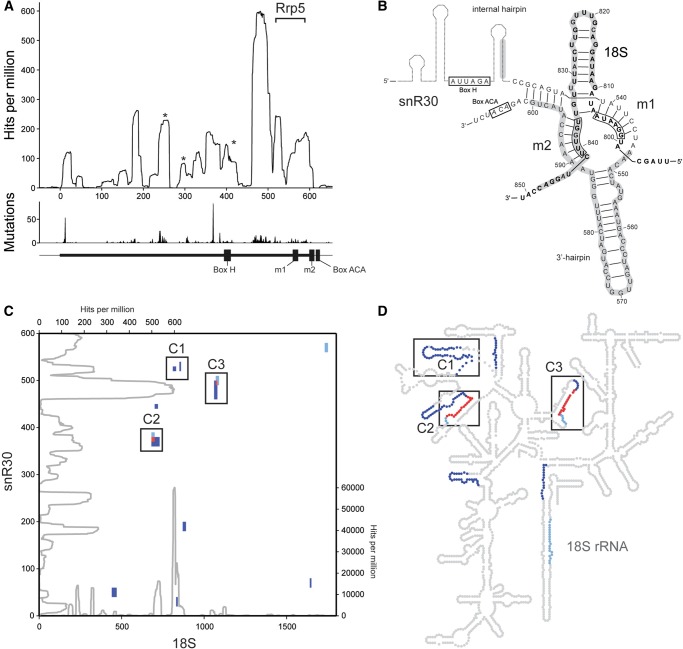
snR30 directly interacts with Rok1 and has additional base-pairing sites in the 18S rRNA sequence. (*A*) Rok1-N-HTP reads that mapped to snR30 are shown as the number of hits for each nucleotide per million mapped reads. Asterisks indicate peaks also found in control samples. Mutations arising during reverse transcription at cross-linked nucleotides are shown in the *lower* panel. Black bars *below* indicate positions of conserved sequence elements and known pre-rRNA interaction motifs. The bracket indicates the position of the previously identified Rrp5-binding site. (*B*) Secondary structure model of interactions between ES6H3 of 18S (bold) and snR30 (data adapted from Fayet-Lebaron et al. 2009). Rok1 cross-linking sites on both RNAs are indicated by gray shading. snoRNA interaction motifs on the 18S rRNA are marked by black boxes. (*C*) The positions of predicted base-pairing sites (light blue) and chimeric reads identified by CLASH (dark blue) are mapped according to their positions on 18S rRNA (horizontal) and snR30 (vertical) and sites of overlap between predicted base-pairing sites and CLASH hybrids are shown in red. Predicted interaction sites and hybrids from CLASH were clustered (black boxes, C1–C3) based on their relative positions in the linear sequence and 18S rRNA structure. The profiles of Rok1 cross-linking on both RNA sequences are shown in gray with the peak heights given for 18S on the *right* and for snR30 at the *top* (see also Figs. 1B, 3A). (*D*) Data presented in *C* were mapped onto the 2D structure of 18S rRNA. Colors are as in *C* and clusters defined in *C* are marked with numbered black boxes.

We have previously shown that Rok1 is required for the release of the snR30 snoRNA from pre-ribosomal complexes (Bohnsack et al. 2008). Since snR30 base-pairs with the 18S rRNA sequence in ES6 (at sites rm1 and rm2), this provides independent evidence for Rok1 binding and functioning in this region of the pre-ribosome. Furthermore, the overlapping binding sites of Rok1 and snR30 may indicate a direct role for Rok1 in regulating the association of snR30. Therefore, to gain more insight into the interactions of Rok1 with this snoRNA–pre-rRNA duplex, we first ascertained, by analysis of our CRAC data, that snR30 strongly cross-links to Rok1. We then analyzed the distribution of the Rok1 cross-linking sites along this snoRNA and found several (and corresponding point mutations in the sequence reads) along the length of the snoRNA (Fig. 3A). Interestingly, the majority of peaks were localized at the 3′ end of the snoRNA where the functionally essential elements of this snoRNA are found (Atzorn et al. 2004; Fayet-Lebaron et al. 2009). Significant cross-linking of Rok1 to the internal hairpin that is structurally important for snR30 function was observed. The m2 motif of snR30 was also identified as a prominent Rok1 interaction site. This correlates well with our identification of the corresponding 18S rRNA sequence, rm2, as a Rok1 binding site (Fig. 3B) and could suggest that Rok1 mediates the release of snR30 by local strand unwinding. When bound to snR30, ES6H3 is suggested to form a hairpin structure (Fig. 3B; Fayet-Lebaron et al. 2009). Since Rok1 is proposed to unwind RNA duplexes with a single stranded extension (Garcia et al. 2012), it is possible that Rok1 mediates unwinding of the snR30–ES6H3 duplex from a binding site in the loop of this hairpin. Furthermore, the cross-linking sites of Rok1 and Rrp5 on snR30 are located in close proximity, which is in line with the adjacent binding sites of these two proteins in ES6H3 and ES6H2 of 18S rRNA, respectively.

Due to its base-pairing interactions with ES6H3, the release of snR30 from pre-ribosomal particles is a prerequisite for formation of the interactions between ES6H3 and ES3 that are found in the mature ribosome (Alkemar and Nygard 2003; Ben-Shem et al. 2011). As the Rok1 helicase contacts all three RNA sequences involved and is required for pre-ribosomal release of snR30, it may function as a key regulator of this structural rearrangement. Furthermore, both ATP-dependent unwinding and ADP-dependent annealing functions of Rok1 have been described (Garcia et al. 2012; Young et al. 2013), possibly implicating this helicase in formation of base-pairing interactions between ES6 and ES3 after the release of snR30 from ES6.

### Novel snR30 base-pairing sites on the 18S rRNA

It has recently become clear that the extent of interactions between snoRNAs and rRNA has been significantly underestimated; additional base-pairing sequences of snoRNAs involved in both rRNA modification and processing have been identified (Kudla et al. 2011; van Nues et al. 2011). We therefore speculated that snR30 might also form additional base-pairing interactions with the 18S rRNA. To investigate this hypothesis, we employed two complementary approaches. Firstly, computational prediction of snR30 base-pairing sites in the 18S rRNA sequence was performed. Secondly, using the cross-linking, ligation and sequencing of hybrids (CLASH) (Kudla et al. 2011) method we analyzed chimeric reads, which arise due to ligation, and thus hetero-concatemerization, of cross-linked RNA fragments or by strand switching of the reverse transcriptase on cross-linked RNA duplexes. Base-pairing prediction has the advantage of providing an unbiased and sensitive search, while chimeric sequences in CRAC data provide experimental evidence of snoRNA–pre-rRNA interactions at the binding sites of proteins of interest.

For the prediction of snR30–18S rRNA hybrids, the sequences of both RNAs were divided into short, overlapping fragments and screened for potential base-pairing interactions using scripts including the RNAduplex algorithm (ViennaRNA package) (Lorenz et al. 2011), and only hits above a minimum stability threshold were considered significant (see Materials and Methods). This approach identified three potential base-pairing sites between snR30 and 18S rRNA while, excitingly, CLASH analysis of the deep sequencing data derived from our Rok1 CRAC experiments revealed hybrids corresponding not only to the known snR30 site in ES6H3 but also additional putative interactions sites in ES6H1, ES7, H15, H22, and H44 of 18S rRNA (Fig. 3C,D). The sites identified by these two independent approaches were overlaid graphically according to their positions on the linear RNA sequences of snR30 relative to 18S (Fig. 3C) and also mapped onto a secondary structure map of 18S rRNA (Fig. 3D). In addition to the known snR30 base-pairing sites in ES6H3 (C1), this excitingly revealed two putative new snR30 interaction sites (C2 and C3), supported by the identification of overlapping CLASH hybrids and predicted base-pairing sites on both 18S rRNA and snR30 (Fig. 3C,D). Firstly, we find a prediction-CLASH hybrid pair that overlap both on snR30 and in ES6H1 of the 18S rRNA (C2). Secondly, our base-pairing prediction analysis also highlighted a very stable, 19-nt sequence in ES7 as a potential snR30 base-pairing site that is supported by the identification of an overlapping CLASH hybrid (C3). This potential interaction is of particular interest because it involves both the snR30 sequence most frequently cross-linked by Rok1 and also a putative Rok1 contact site in the 18S rRNA sequence. Furthermore, this potential base-pairing site overlaps with a recently identified interaction site of the U3 snoRNA in ES7 (Kudla et al. 2011). In addition, base-pairing prediction analysis of *Schizosaccharomyces pombe* snR30 and 18S rRNA also identified a likely interaction (minimum free energy −16 kcal/mol) between ES7 of the 18S rRNA sequence (nucleotides 1051–1070) and a region of the snR30 internal hairpin (nucleotides 226–245) also identified in *S. cerevisiae*. This implies that this potential interaction site of snR30 is likely conserved in other fungi.

### Rok1-associated snoRNAs form multiple interactions with the 18S rRNA

**FIGURE 4.**
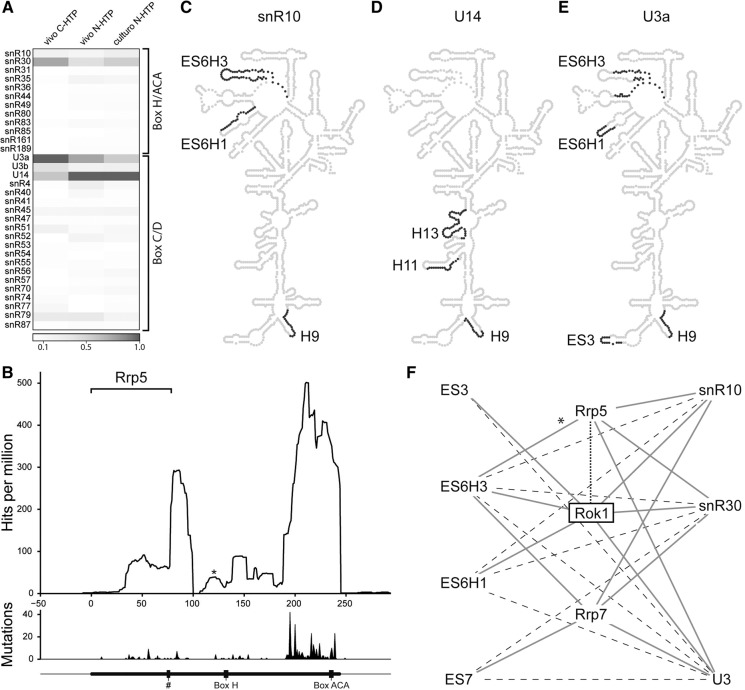
CLASH hybrid analysis identifies novel 18S rRNA base-pairing sites for snR10, U14, and U3. (*A*) Heatmap showing the relative cross-linking of Rok1 to snoRNAs of the SSU. (*B*) Rok1-N-HTP reads that mapped to snR10 are shown as the number of hits for each nucleotide per million mapped reads. The asterisk indicates a peak also found in control samples. Mutations arising during reverse transcription at cross-linked nucleotides are shown in the *lower* panel. Black bars *below* indicate positions of conserved sequence elements and the hash indicates the position of the functionally important 7-nt sequence of snR10. The bracket indicates the position of the previously identified Rrp5 interaction site on snR10. (*C*–*E*) The positions of identified CLASH hybrids on the secondary structure of 18S rRNA are shown for snR10 (*C*), U14 (*D*), and U3a (*E*). (*F*) Interaction network of relevant 18S rRNA regions (*left*), snoRNAs (*right*), and proteins (Rok1, Rrp5, and Rrp7; *center*). Dotted line indicates direct protein–protein interaction, solid lines mark RNA–protein interactions identified by CRAC, and dashed lines denote snoRNA–pre-rRNA interactions detected by CLASH. The asterisk indicates that Rrp5 interacts with an 18S rRNA sequence adjacent to, but not within, ES6H3.

Besides snR30, pre-rRNA interaction sites of other essential snoRNAs have also been described; U3 interacts with 18S rRNA sequences that ultimately form the central pseudoknot as well as with sites in the 5′ external transcribed spacer (ETS), while U14 base-pairs at its modification site in H13 and also in H6a. Less is known about the base-pairing sites of snR10 as the short stretches of base-pairing interactions that box H/ACA snoRNAs form with the pre-rRNA often make their bioinformatic identification challenging. Although a binding site in the 5′ ETS has been reported (Liang et al. 2010), potential interactions of snR10 with the 18S rRNA remain elusive. Interestingly, a significant enrichment of U14, U3a, and to a lesser extent, snR10 sequences was found in our Rok1 CRAC analysis, implying that, as well as snR30, Rok1 might interact with these snoRNAs (Fig. 4A). This raised the possibility that, like snR30, these snoRNAs may base-pair with the pre-rRNA at Rok1 binding sites.

Although Rok1 did not appear to interact with snR10 as strongly as with snR30, U3a, or U14, genetic interactions between snR10 and Rok1 have previously been reported indicating a functional link (Venema et al. 1997). Analysis of the distribution of Rok1 hits along snR10 revealed two peaks: one adjacent to an essential 7-nt sequence (Liang et al. 2010) and the Rrp5-binding site (Lebaron et al. 2013) and a second at the 3′ end of the snoRNA where other functionally important motifs such as the ACA box are found (Fig. 4B). This indicates that Rok1 may be a regulator of snR10 function, so we used CLASH to determine if snR10 base-pairs with 18S rRNA at any of the Rok1 binding sites. This uncovered two potential snR10 interaction sites in ES6H1 and ES6H3 (Fig. 4C); however, CLASH hybrids between ES6H3 and snR10 were not recovered as frequently as those arising between ES6H3 and snR30 described above. Interestingly, snR10 was recently reported to interact strongly with Rrp7 and, based on the binding of Rrp7 to ES7, an snR10 base-pairing site in this region was proposed (Lin et al. 2013). Since Rrp7, like Rok1, also binds to ES6H3, it is tempting to speculate that snR10, possibly regulated by Rok1 or Rrp7, bridges interactions between ES6H3 and ES7 in pre-ribosomal complexes.

For U14, the CLASH analysis identified multiple hybrids containing the previously reported U14 base-pairing and modification site in H13 (Fig. 4D). However, additional hybrid sequences overlapping with the Rok1 cross-linking sites in H9 and H11 were also found. Rrp5 interacts with the 18S rRNA sequence in between these two binding sites (Lebaron et al. 2013), again suggesting an interplay of these factors within these regions of the pre-ribosome.

We also detected hybrids between the U3 snoRNA and ES6H3, ES6H1, ES3, and H9 of the 18S rRNA sequence (Fig. 4E). Depletion of Rok1 has been shown to cause some accumulation of U3 in pre-ribosomal complexes (Bohnsack et al. 2008), which might imply a functional relevance of these interactions. It is important to note that the known U3-binding sites in the 18S rRNA sequence or the 5′ ETS do not overlap with the major Rok1 binding sites. We were therefore unlikely to identify these sequences in our analysis. Interestingly, the discovery of potential U3 base-pairing sites in both ES3 and ES6H3/1 raises the possibility that the U3 snoRNA bridges long-range interactions between these sequences in pre-ribosomes. These interactions, possibly regulated by Rok1, might contribute to folding of the pre-rRNA and help establish the base-pairing between ES3 and ES6, which is found in mature ribosomes.

RNA folding and compaction of early pre-ribosomal complexes into “terminal knobs” can be observed in Miller spreads (Osheim et al. 2004), and recently Rrp5 was proposed to function as a scaffold, coordinating pre-rRNA cleavages and assembly of pre-ribosomes (Lebaron et al. 2013). In concert with this proteinaceous framework, snoRNAs are emerging as additional players in establishing structures within pre-ribosomal complexes that are essential for maturation. On the one hand, interactions between snoRNAs and pre-rRNA are likely important for maintaining an open structure of early pre-ribosomes that is required for rRNA modification by other snoRNPs and the association/action of many biogenesis factors. On the other hand, snoRNAs have important functions in coordinating pre-rRNA cleavages, likely acting in local RNA folding and bringing together distant regions of the transcript. More specifically, snR30, snR10, and U3a base-pairing sites appear to cluster in both ES7 and ES6 (Kudla et al. 2011; Lin et al. 2013), suggesting that these regions might be tethered together in pre-ribosomal complexes. Interestingly, some of the new base-pairing sites of these snoRNAs are adjacent or even overlapping, perhaps indicating sequential association of the snoRNAs. Indeed, it is possible to imagine a scenario in which ES6 is first structurally linked to ES7 by several snoRNAs and that subsequent snoRNA-mediated action brings together ES6 and ES3 to establish the structure found in mature ribosomes. We therefore propose a snoRNA–pre-rRNA–protein interaction network that orchestrates the remodeling of pre-ribosomal complexes (Fig. 4F). As an active ATP-dependent RNA helicase Rok1 is likely to provide the catalytic activity required to drive some of the early transitions in the structure of the pre-ribosome. Interestingly, Rrp5 is one of the few ribosome biogenesis factors that is implicated in the maturation and in fact contacts rRNAs of both ribosomal subunits (Venema and Tollervey 1996; Lebaron et al. 2013). This raises the possibility that the snoRNAs involved in the pre-SSU scaffold might not only coordinate rearrangement of the early pre-SSU but also form contacts to pre-rRNAs of the LSU, thereby coupling maturation of both ribosomal subunits.

In prokaryotes, ribosomal proteins themselves largely coordinate maturation and structuring of the ribosomal subunits while the greater size, complexity, and number of rRNA modifications of eukaryotic ribosomes appear to necessitate the evolution of the large number of ribosome biogenesis cofactors and the formation of a scaffold of snoRNAs and proteins to regulate ribosome assembly. One of the major differences between prokaryotic and eukaryotic rRNAs is the presence of eukaryotic expansion segments, and intriguingly most of the newly identified snoRNA–pre-rRNA interactions revealed here cluster in these regions, suggesting that they may act as important anchor points from which eukaryotic pre-ribosome remodeling is coordinated.

## MATERIALS AND METHODS

### UV cross-linking and analysis of cDNA (CRAC)

For CRAC experiments plasmids based on pRS415 that enable expression of either N- or C-terminally His_6_-Tev-ProteinA (HTP)-tagged Rok1 were transfected into a Rok1 depletion strain, YMB146 (Bohnsack et al. 2008). Where necessary, Rok1 was depleted by addition of 20 µg/mL doxcycline for 9 h. The growth rates of all strains were determined as previously described (Bohnsack et al. 2008). CRAC experiments and analysis of the deep sequencing was performed as previously described (Bohnsack et al. 2009; Granneman et al. 2009; Bohnsack et al. 2012). In brief, UV cross-linking was performed in living cells either in culture medium (in culturo) or pelleted and resuspended in small volume (in vivo), and Rok1-containing complexes were isolated first on IgG sepharose, eluted with TEV protease, and purified on Nickel-NTA. After RNase trimming and linker ligation, fragments were amplified by RT-PCR and sent for Illumina deep sequencing. Mapping of cross-linking data onto the 18S rRNA secondary structure (Petrov et al. 2013) was performed by using Python (Version 2.7) scripts and vector-graphics from http://apollo.chemistry.gatech.edu/RibosomeGallery. Mapping of cross-linking data on the 3D structure of the SSU (Ben-Shem et al. 2011) was performed using Python scripts and Pymol. The height of all peaks relative to the highest peak was calculated and they are mapped in a color gradient of yellow (5%) to red (100%).

### Isolation of Enp1-containing pre-ribosomal complexes

For purification of pre-ribosomal complexes chromosomally encoded Enp1 was C-terminally HTP-tagged in the YMB146 strain. Exponentially growing cells were harvested and lysed by grinding in liquid nitrogen. Enp1-HTP-containing pre-ribosomal complexes were isolated in a one-step purification on IgG sepharose before isolation of co-precipitated RNA using phenol-chloroform. Northern blot analysis was performed using a probe hybridizing to the 5′ end of ITS1 (5′-CGGTTTTAATTGTCCTA-3′). For analysis of the protein composition of purified pre-ribosomal particles, a sample of the pulldown eluate was separated by SDS-PAGE and analyzed by Western blotting using anti-HA (Rok1; Sigma-Aldrich), anti-PAP (Enp1; Sigma-Aldrich), and anti-GAPDH (Abcam) antibodies.

### In vivo chemical probing

DMS structure probing using the Rok1 depletion/Enp1-C-HTP strain was performed as previously described (Swiatkowska et al. 2012). Briefly, exponentially growing cells were depleted of Rok1 for 9 h before pelleting and resuspension in 25 mL YPD medium. DMS was added to a final concentration of 1% for 4 min at 30°C before quenching with 0.7 M ß-Mercaptoethanol in isoamylalcohol and washing with water. RNA was isolated from pre-ribosomal complexes purified via Enp1-HTP as described above. Primer extension using a radiolabelled primer (5′-CGTCCTTGGCAAATGC-3′) was performed using Superscript III reverse transcriptase (Life Technologies) according to the manufacturer's instructions. RNA was separated on a 10% denaturing, polyacrylamide sequencing gel and visualized using a phosphorimager. A sequencing ladder was prepared using the above oligonucleotide, a pAV162 plasmid containing the 35S rDNA as a template and the Sequenase Version 2.0 DNA Sequencing Kit (Affymetrix) according to the manufacturer's instructions.

### CLASH analysis and hybrid predictions

CLASH analysis of hybrids formed during the ligation step of the CRAC procedure was performed as previously described (Kudla et al. 2011; Travis et al. 2013). In brief, sequences were pre-processed using Flexbar (trim = 0, filter = 0, minimum = 4, length = 17) and reads were mapped using BLASTALL. The computational snoRNA–rRNA interaction prediction algorithm was based on the RNAduplex software (ViennaRNA package; Lorenz et al. 2011) and was adapted into a Python script designed to test the base-pairing properties for 20-nt RNA fragments with a 15-nt overlap. A cutoff of −20 kcal/mol minimum free energy was used as a threshold to define stable hybrids. Clustering of experimentally obtained hybrids and predicted base-pairing sites was performed with Python scripts and matplotlib (Hunter 2007).
